# Case report: Open water swimming as a possible treatment for asthma

**DOI:** 10.3389/fmed.2023.1169639

**Published:** 2023-05-05

**Authors:** Kirsty Greenfield, William Verling, Thomas Larcombe, Gary James Connett

**Affiliations:** ^1^Primary Care Practice, Cheviot Road Surgery, Southampton, United Kingdom; ^2^Department of Paediatrics, Dorset County Hospital, Dorchester, Dorset, United Kingdom; ^3^Southampton Children's Hospital, University Hospital Southampton NHS Foundation Trust, Southampton, United Kingdom; ^4^National Institute for Health Research, Southampton Biomedical Research Centre, Southampton Children's Hospital, Southampton, United Kingdom

**Keywords:** open water swimming, asthma, exercise induced asthma, seasonal asthma, cold water shock, diving reflex, involuntary panting

## Abstract

Asthma is a complex medical problem for which currently available treatment can be incompletely effective. This case report describes a 49 year old woman who had suffered from asthma since her teenage years that resolved after she took up regular open water swimming. After sharing this case report with an international open water swimming community on social media, over one hundred people with asthma commented that their symptoms had also improved after taking up this activity. The mechanism whereby open water swimming might alleviate asthma has not been established. Possibilities include benefits to mental health, anti-inflammatory effects, being more fit, improved immune function and suppression of the bronchoconstrictive component of the diving reflex. Further research might usefully confirm or refute these clinical observations.

## Introduction

Regular exercise can improve quality of life for people with asthma (1). This occurs through a number of mechanisms including increased strength, lung capacity and cardiovascular fitness. Improvements in bronchial hyperresponsiveness after exercise is significantly associated with improvements in asthma symptoms (2). As a result, regular physical activities are often recommended as supplementary therapy to the use of medication.

Swimming has been recommended for many years as a good form of exercise for asthmatics (3) although more recently there is emerging evidence that it can increase symptoms and markers of airways inflammation when carried out in both indoor and outdoor public pools (4). This is thought to occur as a result of inhaling the chloramines that are used to disinfect pool water (5). At the elite level, aquatic sports are associated with more asthma than other endurance pursuits (6).

There is very little literature on the effects of open water swimming on asthma, but there is growing evidence for other health benefits including anti-inflammatory effects on arthritis, improved immune function and improvements in mental health (7, 8). There are several potential mechanisms whereby such effects could be occurring. These include social and psychological benefits as well as those occurring more directly on cellular function. All such mechanisms might be relevant to the occurrence and severity of asthma. This case report is about an adult with chronic asthma that resolved after regular open water swimming.

## Case description

**Figure 1:**
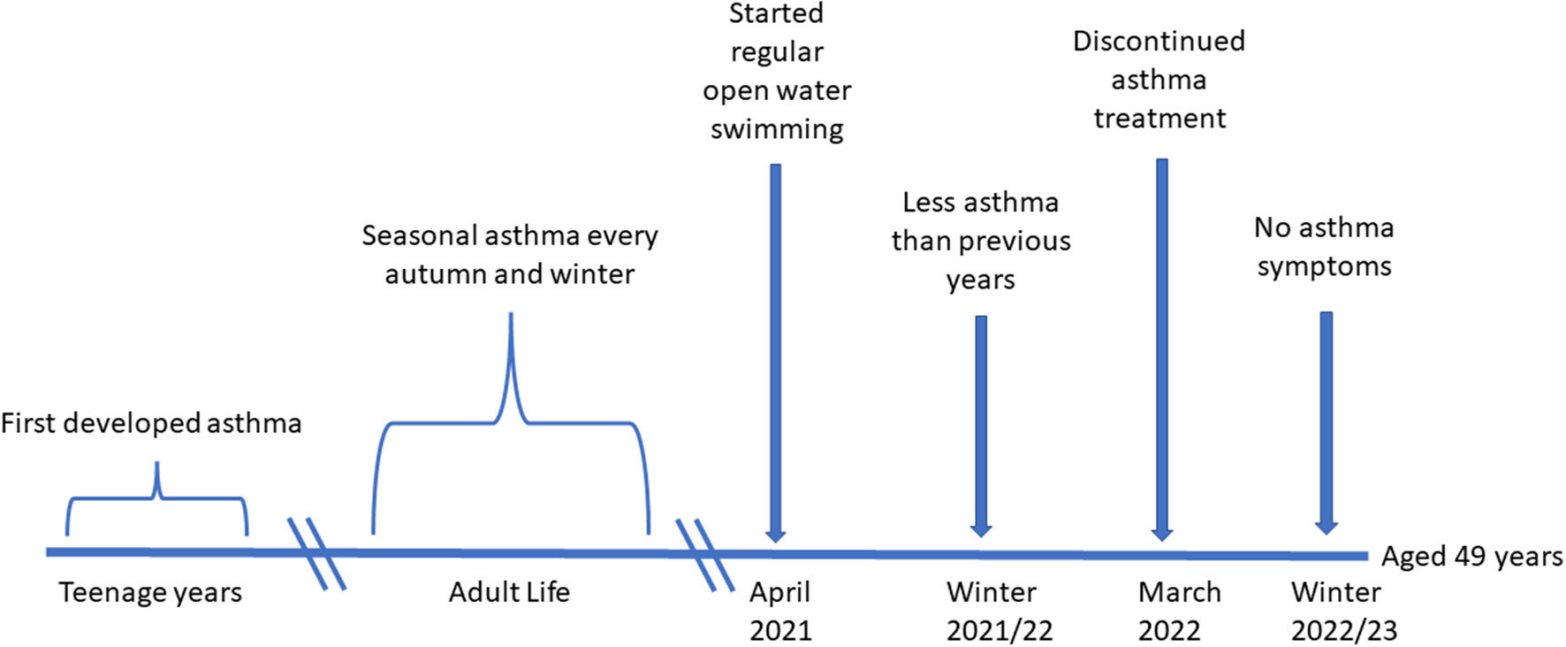
Time Line.

The case report is of a 49 year old woman (Figure 1). She first developed asthma in her early teens. Her symptoms at that time were cough and wheeze after exercise or cold air inhalation and nocturnal cough. She had suffered episodes of acute severe wheeze, with decreased peak expiratory flow rates down to 50% of predicted, and these were treated with up to 10 puffs of salbutamol *via* a spacer 4 h. Treatment had been effective in alleviating symptoms without the need for more intensive treatment in hospital.

Throughout her adult life she had suffered seasonal cough, wheeze, chest tightness and shortness of breath at the onset of damp autumn weather. This had occurred despite taking regular inhaled corticosteroids at this time of the year. She inhaled salbutamol as needed to help alleviate symptoms. Seasonal upper respiratory tract infections caused worsening wheeze for which increased doses of salbutamol were needed for 1–2 weeks on each occasion. Symptoms were typically worse after exercise and in cold weather and continued each year until around Easter.

Treatment was with regular beclomethasone 100 mcg MDI 2 puffs twice daily commenced every autumn and continued until the late spring. Double doses were given at the onset of acute respiratory illness. Trials of regular combination inhalers using the long-acting beta-2 agonists salmeterol and formoterol were not continued. They were not well tolerated and caused tremor and palpitations. Antihistamines and mometasone nasal spray had been prescribed for perennial rhinitis. When used during some of the worse months for asthma, they seemed to help improve respiratory symptoms.

Her past medical history included contact dermatitis and urticarial rashes after exposure to particular detergents, plants, and dyes. She had a hysterectomy in 2020 to treat fibroids and had been treated since then with Evorel 75 mcg hormone replacement patches (HRT). She had been prescribed Sertraline 50 mg once daily in September 2021 and this was reduced to 25 mg in March 2022. She was a lifetime non-smoker and drank approximately 2 units of alcohol per week. She was a fit regular cyclist but had to use bronchodilators to prevent and treat exercise induced asthma in relation to this activity during winter months. She had a pet spaniel in the house over the previous 5 years and ate a healthy mixed diet.

## Investigation, intervention and outcome

The patient provided a clear medical history consistent with a diagnosis of asthma. Her Peak Expiratory Flow Rate (PEFR) when well was usually between 370 and 390L/min and below the mean predicted level of 400 L/min for her age and height. She had measured >20% reductions in baseline PEFR on multiple occasions with improvements after inhaling salbutamol. Her body mass index was normal at 22.0 and blood pressure 126/80. She did not have eosinophilia on peripheral blood testing and there was no evidence of allergic sensitisation to common aeroallergens. This history and the clinical findings were entirely consistent with the diagnosis of asthma with no indication that there was an alternative cause for recurrent wheezing.

She commenced regular open water swimming in the sea at least once a week in April 2021. She maintained a regular swimming routine and only missed a couple of weeks each year at times of viral infection. Between June and September she tended to swim more often than weekly. She swam in a swimsuit plus neoprene gloves, hat, and socks. Water temperatures were at an average low of 7.2°C in February, rising gradually throughout the year to an average maximum of 18.7°C in August. Time spent swimming was tailored to the water temperature increasing from 8 min when 7–8°C, to 10–12 min when 9–10°C, to 12–16 min when 11–14°C and for at least 20 min when the water temperature was above 15°C.

During the 2021–2022 winter season her asthma was less problematic. She still experienced occasions when she needed to take relief medication but the frequency and the number of inhalations needed were far less than in previous years. At the end of March 2022, she discontinued regular inhaled corticosteroids for her seasonal symptoms. Given the improved symptoms over the previous winter, and the complete absence of any further asthma symptoms over the following months, she did not restart inhaled steroids the following autumn. Over the 202–23 winter season she experienced a remarkable improvement in her health and was able to exercise and be outside in cold air without any chest symptoms and without the need to take salbutamol. Although she had significant upper respiratory tract infections as in previous winters, these did not result in wheeze and the need for bronchodilators.

Resolution of asthma symptoms has been sustained without the need for asthma medication. There have been no new emerging health care problems or any medical concerns in relation to the continuation of open water swimming. Approximately 12 months after stopping all asthma medication and 26 months after commencing her swimming routine, the results of spirometry were FEV1, 3.18L (110% of predicted), FVC, 4.27L (118% of predicted) and MMEF75/25, 2.49L/s (88% of predicted). Exhaled FeNO was 27 ppb: just above the normal range of < 25 ppb.

## Discussion

Asthma is a complex disease with no consistent gold standard diagnostic criteria (9). Although spirometry and FeNO measurements were not obtained prior to open water swimming, the history of recurrent wheezing and evidence of reversible airflow obstruction as documented by PEFR measurements before and after inhaled bronchodilators, suggest the clinical diagnosis was correct.

This is the first case report of an asthmatic whose symptoms have resolved after open water swimming. This is perhaps surprising as inhalation of cold air, as would have occurred during this activity, is often reported as an asthma trigger and a cause of worsening symptoms in those with poor control (10).

As her symptoms were only a problem during autumn and winter, she had elected to only take her regular preventer treatment during this time of the year. Whilst this is a reasonable approach to treatment, underlying airways inflammation might still have been occurring throughout the year, even when symptoms were not present, and result in airway remodeling (11). The normal lung function results suggest that this had not occurred.

There are several possible explanations for the resolution of asthma symptoms as described.

Breathing in a salty environment is of benefit to children with cystic fibrosis as a result of improved airway clearance (12). However, increasing concentrations of inhaled hypertonic solutions can also cause bronchoconstriction and in particular in those with asthma. Speleotherapy, breathing air in cave environments which often have a high salt concentration, is practiced in many parts of the world to treat people with chronic medical conditions including asthma. There is some limited evidence for the benefits of this practice although the mechanism whereby this occurs is not understood (13).

Steroid naive patients with asthma participating in randomised controlled trials of inhaled corticosteroids typically have significant placebo benefits. Improvements in lung function can be up to 50% of that achieved by participants receiving active treatment (https://www.gsksource.com/floventdiskus). Patient benefits as a result of belief in the likelihood of receiving an effective treatment or intervention, combined with high quality supportive care, are important components of good asthma management (9). It is possible that asthma could improve through similar placebo effects after open water swimming. This seems unlikely however because the activity was taken up with no expectation of benefits to respiratory health.

There is a clear association between anxiety and depression and worsening asthma and there is emerging evidence that open water swimming can positively impact on mental health outcomes (8, 14). There are several potential mechanisms whereby this could occur. These include boosting dopamine levels and the release of endorphins, spending time outdoors in blue and green spaces, becoming a part of a wild swimming community and the resilience that might come with overcoming the resistance to entering cold water. Although our case had recently started treatment with Sertraline and their mental health had been better than average when their asthma improved, this was not thought to be the entire explanation. The patient observed that her mental health status had fluctuated throughout her adult life and there was no relationship between this and the consistent seasonal pattern of her asthma. Her improvement was unlikely to have been due to the communal benefits of joining in with others as she swam by herself under the supervision of her partner. She reported that her blue green exposure had not changed as a result of open water swimming. She had regularly cycled or walked in green and blue spaces for a minimum of 4 h per week for many years.

Regular exercise can be of benefit to asthmatics, but this is unlikely to have resolved symptoms in this case. Activity and fitness levels had been consistently high prior to open water swimming and 10 years previously she had done pool based triathlon training with no improvement in her asthma. She continued all her other physical activities in the same way as she had prior to taking up open water swimming.

Our patient had a hysterectomy and started oestradiol HRT in the year prior to starting open water swimming and she has remained on this treatment. There is a complex relationship between the menopause, asthma, and HRT. Studies suggest that a lack of oestrogen is associated with improvements in asthma. Continuation of oestrogen HRT can worsen asthma whereas progesterone replacement can be protective (15). In this case oestradiol HRT without progesterone was taken which would have been more likely to result in continuation of asthma symptoms.

Less frequent and less severe winter viruses is often cited as an anecdotal benefit of cold water swimming and there is emerging evidence for positive effects on immune function (7, 16). Whilst this could have been a factor in this case, she had 2 colds over the recent winter months, and these did not result in any asthma symptoms as had invariably happened previously. Alterations in immune responsiveness to prevent airways inflammation and increased airways hyperresponsiveness might also have occurred, although no potential mechanisms for this have been investigated.

Immediately after immersion in cold water there is a cold water shock response. The most obvious feature of this is involuntary gasping (7). This is a parasympathetic reflex to which there is adaptive change after repeat immersions. A 50% reduction in the physiological response to cold water < 15°C in temperature has been shown to occur after just 6 immersions. This adaption has been shown to last up to 7–14 months (17). Some researchers have emphasized the importance of the autonomic nervous system in relation to asthma and have reported that increased parasympathetic vagal responsiveness is associated with more severe symptoms (18). Such effects are consistent with the effectiveness of anticholinergic drugs when used in addition to maximal doses of beta-2-agonists in the treatment of severe asthma attacks (19). It is possible that the prolonged down regulation of the cold water shock reflex after repeated cold water immersions might also result in the down regulation of other autonomic reflexes.

The diving reflex is well recognised autonomic reflex in humans and one small study has shown that it is increased in asthmatics (20, 21). An underappreciated part of this reflex is the contraction of smooth airways muscle (22). This has been well characterised in aquatic mammals as a mechanism to prevent nitrogen absorption when diving at depth to prevent the bends (23). It is possible that repeated open water swimming suppresses the reflex vagal contribution to bronchial constriction as well as the cold water shock reflex.

Whilst this is a single anecdotal case, with the permission of the patient, we have shared these findings with the open water swimming community through a popular YouTube channel and Facebook group (https://www.youtube.com/c/everydayathleterach). Over two thousand open water swimmers viewed the video and of these over one hundred commented that they had also experienced marked improvement or resolution of asthma symptoms since taking up the activity. Less than ten reported little or no benefit or referred to the involuntary feeling of breathlessness that occurs after immersion as result of the cold water shock reflex. Only two open water swimmers reported that their asthma was worse and one of these was unlikely to have been swimming in water < 15°C at any time of the year. Nobody reported that they had taken up this activity with the intention of improving their asthma.

It is not yet possible to make any recommendations in relation to open water swimming and improving asthma symptoms. The temperature of water is highly variable in different parts of the world, in different open water environments and varies throughout the year. To what extent the amount of time spent submerged and swimming and whether wearing a wet suit impacts on any potential benefits is also not known. It is important to note that the prevalence of asthma in aquatic sports amongst elite athletes is higher than most other sports. These findings suggest that contrary to the warm humid environment of the swimming pool preventing the occurence of asthma symptoms, the opposite is more likely. This is probably due to prolonged training in a chlorinated environment causing pro-inflammatory effects on airway epithelium and smooth muscle. The prevalence of asthma is also high in open water swimmers at the elite level although it is likely that many of these athletes train some of the time in chlorinated pools. Within the aquatic sports the endurance disciplines are associated with more asthma than non-endurance disciplines. This could be due to the inflammatory effects of prolonged breathing at high volumes and respiratory rates. Such effects would occur in endurance swimmers irrespective of the environment in which they were training but are less likely to be an issue for recreational swimmers (6). In this case symptoms did not resolve because of switching from a chlorinated pool to open water swimming. She continued to swim weekly in a pool, as she had for many years, and added in regular open water swimming.

The anecdotal recovery of asthma after open water swimming as reported and the feedback from the open water community is of interest and warrants further investigation. Research in this area might usefully result in a better understanding of the important determinants of asthma and the development of more effective treatment.

## Patient's perspective

It has been liberating for me to breathe the cold winter air this year with no cough or wheeze and no dependency on the inhalers I have needed since my early teens. Although my asthma has never been severe, this change feels unprecedented for me. This is a fascinating area in which I would love to see further research. However cold open water swimming is not without risk and anyone considering it needs to carefully think through the safety implications of what they are planning, acclimatise gradually and take advice from experienced swim leaders and/or medical professionals as appropriate.

## Data availability statement

The original contributions presented in the study are included in the article/supplementary material, further inquiries can be directed to the corresponding author.

## Ethics statement

Written informed consent was obtained from the individual(s) for the publication of any potentially identifiable images or data included in this article.

## Author contributions

GC assessed the patient and led the writing of the manuscript. TL assessed the patient and provided feedback in developing the manuscript. WV identified the case, provided expertise in relation to open water swimming literature, and assisted in writing the manuscript. KG provided clinical details and helped write the manuscript. All authors contributed to the article and approved the submitted version.
